# Histopathological Clues of Enhanced Inflammation in the Placental Tissue of Women with Chronic Venous Disease in Lower Limbs during Pregnancy

**DOI:** 10.3390/jpm14010087

**Published:** 2024-01-12

**Authors:** María Asunción Sánchez-Gil, Oscar Fraile-Martinez, Cielo García-Montero, María Del Val Toledo, Luis G. Guijarro, Juan A. De León-Luis, Coral Bravo, Raúl Díaz-Pedrero, Laura López-Gonzalez, Miguel A. Saez, Melchor Álvarez-Mon, Natalio García-Honduvilla, Miguel A. Ortega

**Affiliations:** 1 Department of Medicine and Medical Specialities, Faculty of Medicine and Health Sciences, University of Alcalá, 28801 Alcalá de Henares, Spain; msangil@oc.mde.es (M.A.S.-G.); cielo.garciamontero@uah.es (C.G.-M.); msaega1@oc.mde.es (M.A.S.); mademons@gmail.com (M.Á.-M.); natalio.garcia@uah.es (N.G.-H.); 2 Ramón y Cajal Institute of Sanitary Research (IRYCIS), 28034 Madrid, Spain; mval.toledo@uah.es (M.D.V.T.); luis.gonzalez@uah.es (L.G.G.); raul.diazp@uah.es (R.D.-P.); laura.lgonzalez@uah.es (L.L.-G.); 3 University Defense Center of Madrid (CUD), 28047 Madrid, Spain; 4 Department of Biomedicine and Biotechnology, University of Alcalá, 28801 Alcalá de Henares, Spain; 5 Department of Systems Biology, Faculty of Medicine and Health Sciences (Networking Research Center on for Liver and Digestive Diseases (CIBEREHD)), University of Alcalá, 28801 Alcalá de Henares, Spain; 6 Department of Public and Maternal and Child Health, School of Medicine, Complutense University of Madrid, 28040 Madrid, Spain; jaleon@ucm.es (J.A.D.L.-L.); coral.bravo@salud.madrid.org (C.B.); 7 Department of Obstetrics and Gynecology, University Hospital Gregorio Marañón, 28009 Madrid, Spain; 8 Health Research Institute Gregorio Marañón, 28009 Madrid, Spain; 9 Department of Surgery, Medical and Social Sciences, Faculty of Medicine and Health Sciences, University of Alcalá, 28801 Alcalá de Henares, Spain; 10 Pathological Anatomy Service, University Hospital Gómez-Ulla, 28806 Alcalá de Henares, Spain; 11 Immune System Diseases-Rheumatology and Internal Medicine Service, University Hospital Prince of Asturias, Networking Research Center on for Liver and Digestive Diseases (CIBEREHD), 28806 Alcalá de Henares, Spain

**Keywords:** pregnancy, women, chronic venous disease (CVD), placenta, allograft inflammatory factor 1 (AIF-1), inflammation

## Abstract

It is estimated that approximately one in three women develop chronic venous disease (CVD) during pregnancy, a broad spectrum of morphofunctional disorders affecting the venous system in different regions of the body, including the lower limbs. A growing body of evidence supports the diverse maternofetal consequences derived from this condition, with the placenta being an organ particularly affected. Among other consequences, having CVD during pregnancy has been associated with systemic inflammation and altered cytokines and chemokine profiles in the maternal and fetal serum related to this condition. In the present work, we aimed to analyze if these inflammatory changes also occurred in the placental tissue of women with CVD, exploring by immunohistochemistry and real-time PCR (RT-qPCR) gene and protein expression of critical inflammatory markers like allograft inflammatory factor 1 (AIF-1), interleukin 10 (IL-10), IL-12A, and IL-18. Our results demonstrate an enhanced tissue expression of AIF-1, IL-12A, and IL-18, accompanied by a decrease in IL-10 in the placentas of women who had undergone CVD during pregnancy. Overall, our results suggest a possible pathophysiological role of inflammation in the placental tissue of women with CVD during pregnancy, although the precise consequences of this feature remain to be deeply analyzed.

## 1. Introduction

Chronic venous disease (CVD) is a condition characterized by functional and structural changes in the venous system caused by ambulatory venous hypertension [1]. It seems that the interaction between genetic and environmental factors is responsible for the increased venous pressure associated with CVD, commonly manifested in the form of varicose veins (VVs) affecting the lower limbs [2,3,4]. It is estimated that one in three women will develop CVD during pregnancy and that women exhibit an increased risk with the number of pregnancies [5]. CVD might appear during pregnancy because of an increase in venous pressure in the lower limbs associated with compression of the inferior vena cava and iliac veins caused by the gravid uterus as well as by the increase in venous distensibility associated with the hormonal changes that occurred in this period [6,7,8]. We have previously evidenced that suffering from CVD during this period can result in a significant stressor for maternofetal structures, with the placenta and the umbilical cord showing evidence of various pathological processes [9,10]. However, the extent and possible pathobiological involvement of these changes in maternofetal structures need to be further characterized. 

Allograft inflammatory factor 1 (AIF-1) is a calcium-binding protein encoded within the HLA class III genomic region [11]. AIF-1 is a molecule tightly linked to inflammatory processes, fulfilling a key role in health and disease conditions [12]. AIF-1 is associated with the activation and function of different cytokines like interleukin 10 (IL-10), IL-12, and IL-18 [13,14]. Previous work has found that CVD is associated with a systemic increase in the levels of circulating proinflammatory markers in both the mother and the fetus [15]. This proinflammatory environment seems to affect different maternofetal structures like the placenta and the umbilical cord, which also evidence significant changes with different inflammatory mediators [14,16]. Therefore, compelling evidence supports the existence of an association between CVD and inflammation that might be affecting both the mother and the fetus. However, the study of this connection and the consequence for maternofetal structures remain to be deeply explored. In this sense, the aim of the present study is to analyze gene and protein expression of AIF-1, IL-10, IL-12, and IL-18 in the placentas of women with CVD by performing real-time quantitative PCR (RT-qPCR) immunohistochemistry. 

## 2. Patients and Methods

### 2.1. Study Design and Participants

A prospective, analytical, and observational investigation was conducted with 114 pregnant women in the third trimester. There were 52 women who had no history of CVD, known as healthy controls (HC), and 62 women who had been diagnosed with CVD according to the CEAP categorization [17]. Using the same samples than in previously published works [9] and as represented in Table 1, women with HC had median gestational ages of 34 (interquartile range (IQR), 27–41 years) and gestational durations of 41 (IQR, 39–42 weeks), whereas women with CVD had median gestational ages of 33 (IQR, 22–40 years) and gestational durations of 40.5 (IQR, 39–41.5 weeks). Regarding the number of prior pregnancies, there were no appreciable differences between the groups: 19 (36.5%) for women in the HC group, and 33 (53.2%) for women with CVD (Table 1). Clinical features such as gestational age, c-section delivery, prior pregnancies, prior abortions, normal menstrual cycles, and type of job (sedentary, Table 1) did not significantly differ between the CVD and HC groups.

Women over the age of 18 with third-trimester medical evidence of venous disease in the lower extremities met the inclusion criteria for our study according to the clinical–etiological–anatomical–pathophysiological criteria (CEAP) ≥ 1 [17]. We excluded from this study women who had previously been diagnosed with high blood pressure; venous malformations; heart, kidney, or lung insufficiency; autoimmune diseases; a BMI of less than 25; diabetes mellitus, gestational diabetes mellitus, or other endocrine conditions; infectious diseases that are active; toxicological habits (use of drugs like cannabis, heroin, cocaine, or amphetamines); preeclampsia and/or HELLP syndrome; alcohol consumption of less than one unit per day; tobacco use of less than one cigarette per day; recognized causes of intrauterine development limits; pathological injuries including placental infarction, avascular villi, delayed villi maturation, or chronic villitis; the appearance of any exclusion criteria in the upcoming months (until delivery); and previous CVD evidence.

The core ethical concepts of autonomy, beneficence, non-maleficence, and distributive justice were followed in the completion of the current work. Additionally, the guidelines for Good Clinical Practice, as well as the values outlined in the most recent Helsinki Declaration (2013) and Oviedo Convention (1997), were adhered to. Prior to enrollment, patients were informed, and each participant gave their own written consent. The Central University Hospital of Defense University of Alcalá’s Clinical Research Ethics Committee granted approval for the current study (37/17). The clinical history was reviewed, and the women’s overall physical health was examined at the third trimester consultation. Additionally, ultrasounds of the lower limbs were carried out at 7.5 MHz with an Eco-Doppler (Portable M-Turbo Eco-Doppler; SonoSite, Inc., Bothell, WA, USA).

### 2.2. Sample Processing

Following delivery, placental biopsies were performed on the 114 patients. Each placenta had five pieces, each with a different mix of cotyledons, that were cut apart using a knife. Two separate sterile tubes containing placental fragments were then filled with RNAlater^®^ (Ambion; Thermo Fisher Scientific, Inc., Waltham, MA, USA) solution and Minimum Essential Medium (MEM; Thermo Fisher Scientific, Inc., Waltham, MA, USA) containing 1% antibiotic/antimycotic (Streptomycin, Amphotericin B, and Penicillin; Thermo Fisher Scientific, Inc.). Following that, the samples were processed under sterile conditions in a class II laminar flow hood (Telstar AV 30/70 Müller 220 V 50 MHz; Telstar; Azbil Corporation, Chiyoda-ku, Tokyo, Japan). The samples were preserved for subsequent processing and gene expression analysis by being placed in 1 mL of RNAlater^®^ and kept at 80 °C. Placentas from MEM that had been preserved were used for histological and immunohistochemical investigations.

To remove the erythrocytes, MEM without antibiotics was used to rehydrate the MEM samples 5 times. Then, they were cut into pieces of 2 cm length and fixed in F13 according to established protocols (60% ethanol, 20% methanol, 7% polyethylene glycol, and 13% distilled water) [9]. After that, molds were used to embed the samples in paraffin. After the paraffin had hardened, sections of 5 m thickness were cut with an HM 350 S rotation microtome (Thermo Fisher Scientific, Inc., Waltham, MA, USA). The sections were subsequently placed in a hot water bath and mounted on glass slides that had priorly been treated with 10% polyLys to improve adhesion.

### 2.3. Gene Analysis through Real-Time Quantitative PCR (RT-qPCR)

RNA was isolated using the guanidinium thiocyanate–phenol–chloroform method, enabling the examination of mRNA expression levels of specific genes [18]. Complementary DNA (cDNA) was synthesized from the RNA samples (concentration of 50 ng/µL) through reverse transcription (RT). During this process, each sample was mixed with an oligo-dT solution and denatured at 65 °C for 10 min. Subsequently, a reverse transcription mix was added to each sample, containing various components such as first strand buffer, deoxyribonucleotides triphosphate, dithiothreitol, DNase- and RNase-free water, RNase inhibitor, and reverse transcriptase enzyme.

The reverse transcription process was completed using a G-Storm GS1 thermal cycler. The samples were heated to 70 °C for 15 min to denature the reverse transcriptase enzyme, before being gradually cooled to 4 °C. The samples were first heated to 37 °C for 75 min to facilitate the synthesis of cDNA. In order to ensure that there was no genomic DNA contamination, a negative reverse transcription was carried out by substituting water devoid of DNases and RNases for the M-MLV RT enzyme. The resultant cDNA was kept at −20 °C after being diluted 1:20 in water devoid of DNases and RNases.

Primer-BLAST and AutoDimer online tools were used to build specific primers for the chosen genes [19,20]. The constitutively expressed TATA-box binding protein (TBP) gene functioned as a normalization control [21]. Relative amounts of mRNA were used to measure the gene expression units. RT-qPCR was carried out, utilizing the relative standard curve method on a StepOnePlus^TM^ System (System (Applied Biosystems; Thermo Fisher Scientific, Inc.). In a MicroAmp^®^ 96-well plate (Applied Biosystems; Thermo Fisher Scientific, Inc., Waltham, MA, USA), the sample (diluted 1:20) was mixed with iQTM SYBR^®^ Green Supermix (Bio-Rad Laboratories, Inc Hercules, California, USA.), forward and reverse primers, and RNase- and DNase-free water for the reaction. Multiple cycles of denaturation, annealing at different temperatures, and elongation made up the thermocycling conditions. For additional examination, a dissociation curve was produced.

Fluorescence detection was carried out during the dissociation curve phases and at the conclusion of each amplification cycle. A combination of the samples was serially diluted in compliance with the manufacturer’s instructions to create a standard curve containing information from the selected genes. The constitutive expression of TBP was then added to each plate along with this curve. The RT-qPCR was performed twice on each placenta tissue sample, following prior work [14] (Table S1).

### 2.4. Immunohistochemical Analysis

The avidin–biotin complex technique using avidin peroxidase was used to detect the antigen–antibody reactions, as previously reported [9]. Immunohistochemical studies were conducted on paraffin-embedded placental samples. The specific antibodies utilized in the study are outlined in Table S2 of the protocol.

The placental samples were exposed to the primary antibody for 90 min before being incubated with 3% BSA Blocker and PBS overnight at 4 °C to prevent nonspecific binding, as is described in [14]. On the next day, the tissues were incubated for 90 min at room temperature with a biotin-conjugated secondary antibody diluted in PBS (Table S2). ExtrAvidin^®^-Peroxidase (Sigma-Aldrich, St. Louis, MO, USA) was then added and allowed to sit at room temperature for 60 min after being diluted 1:200 with PBS.

To determine the protein expression levels, a chromogenic diaminobenzidine (DAB) substrate kit was used (prepared just before use with 5 mL distilled water, two drops of buffer, four drops of DAB, and two drops of hydrogen peroxide). The DAB substrate allowed for the development of a brown stain, facilitating the visualization of the signal. Negative control sections for each protein underwent the same process; however, the primary antibody was replaced with a blocking PBS solution. Hematoxylin staining with Carazzi hematoxylin for 15 min was employed for contrast in all tissues.

### 2.5. Histopathological Examination 

Preparations were inspected using a Zeiss Axiophot optical microscope (Zeiss GmbH). Five portions and ten randomly chosen fields of vision for every patient in the predetermined groups were evaluated. Based on the immunoreactive score (IRS) established in prior investigations, the expression was categorized as positive if the marked mean area in the examined sample was 5% of the total [22,23]. Each sample was given a score on a scale of 0–1 for minimal staining (25%), 2–4 for moderate staining (25–65%), and 3–4 for marked staining (65–100%) by two independent histologists.

### 2.6. Statistical Analysis

Statistical analysis was performed using the GraphPad Prism^®^ v6.0 program (GraphPad, Inc., San Diego, CA, USA). A Kolmogorov–Smirnoff test was used to determine whether the markers were normal (all *p* < 0.001). Since it was evident that the data did not fit into a normal distribution, non-parametric tests were used to describe the data using medians and interquartile ranges. The Mann–Whitney U test was employed to compare the two groups. Significance levels were established as follows: *p* < 0.05 (*), *p* < 0.01 (**), and *p* < 0.001 (***). 

## 3. Results

### 3.1. The Placenta of Women with CVD Exhibits Increased Expression of Proinflammatory Markers AIF-1, IL-12A, and IL-18

We evaluated the expression of proinflammatory markers in the placenta (AIF-1, IL-12A, and IL-18) of women with CVD and compared them with the HC. 

**Figure 1:**
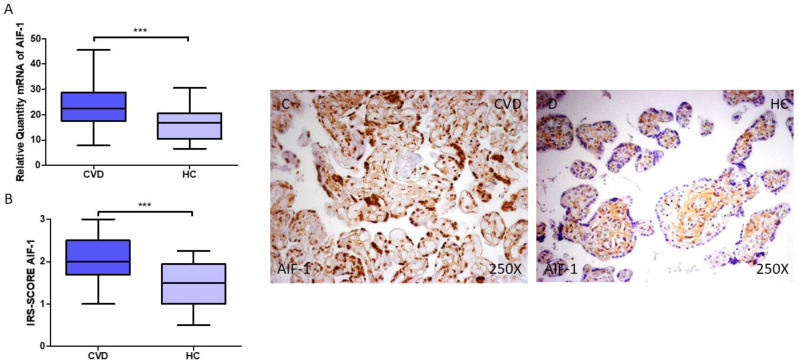
(**A**) RT-qPCR measurements of AIF-1 mRNA expression in the placenta. (**B**) IRS-SCORE protein expression levels for AIF-1 in the placenta. (**C**,**D**) Images showing AIF-1 immunohistochemistry in the placenta. *p* < 0.001 (***).

AIF-1 gene and protein expression in the placenta of women with CVD was investigated first. According to our findings, pregnant women with CVD had higher levels of AIF-1 gene expression in their placental tissue (Figure 1A; relative expression CVD = 22.354 (7.869–45.516), HC = 16.774 (6.616–45.516), *** *p* = 0.0001). Histological examination revealed that women with CVD had significantly higher levels of AIF-1 protein expression in their placentas (Figure 1B; CVD = 2.000 (1.000–3.000), HC = 1.500 (0.500–2.250), *** *p* = 0.0001). According to histopathology pictures, AIF-1 protein expression was considerably higher in women with CVD, indicated by notable differences in the syncytiotrophoblast layer (Figure 1C,D).

**Figure 2:**
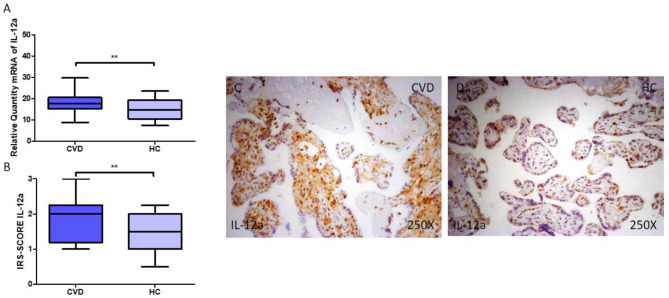
(**A**) RT-qPCR measurements of IL-12A mRNA expression in the placenta. (**B**) IRS-SCORE protein expression levels for IL-12A in the placenta. (**C**,**D**) Images showing IL-12A immunohistochemistry in the placenta. *p* < 0.01 (**).

IL-12A gene and protein expression in the placenta of women with CVD was then explored. We observed that pregnant women with CVD had higher levels of IL-12A gene expression in their placentas (Figure 2A; CVD = 17.714 (8.895–29.785), HC = 14.676 (7.562–23.52), ** *p* = 0.0078). Histological examination demonstrated that women with CVD had significantly higher levels of IL-12A protein expression in the placental tissue when compared to HC (Figure 2B; CVD = 2.000 (1.000–3.000), HC = 1.750 (0.500–2.250), ** *p* = 0.0017). According to histopathology pictures, IL-12A protein expression was notably higher in women with CVD, particularly in the inner layers of the placental villi (cytotrophoblasts, fibroblasts, and other critical cells located in this region) (Figure 2C,D).

**Figure 3:**
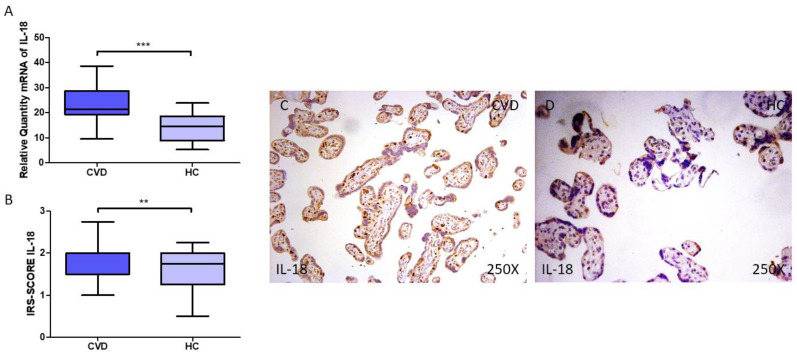
(**A**) RT-qPCR measurements of IL-18 mRNA expression in the placenta. (**B**) IRS-SCORE protein expression levels for IL-18 in the placenta. (**C**,**D**) Images showing IL-18 immunohistochemistry in the placenta. *p* < 0.01 (**); *p* < 0.001 (***).

IL-18 gene and protein expression was evaluated in the placenta of women with CVD. We report that pregnant women with CVD exhibit enhanced levels of IL-18 gene expression in their placental tissue (Figure 3A; CVD = 21.461 (9.562–38.617), HC = 14.504 (5.317–23.900), *** *p* < 0.0001). Histological examination demonstrated that women with CVD had significantly higher levels of IL-18 protein expression in the placental tissue when compared to HC (Figure 3B; CVD = 2.000 (1.000–3.000), HC = 1.750 (0.500–2.250), ** *p* = 0.0017). As indicated by the histopathological images (Figure 3C,D), IL-18 protein expression was notably higher in the placental villi of women with CVD, including in the syncytiotrophoblast and inner layers (Figure 3C,D).

### 3.2. The Placental Tissue of Women with CVD Shows a Decreased Expression of IL-10

**Figure 4:**
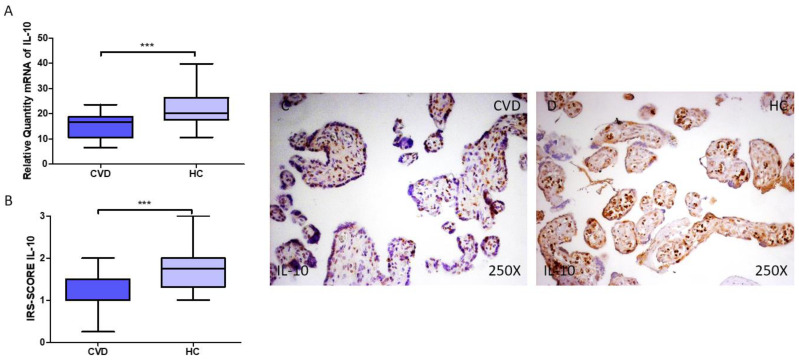
(**A**) RT-qPCR measurements of IL-10 mRNA expression in the placenta. (**B**) IRS-SCORE protein expression levels for IL-10 protein in the placenta. (**C**,**D**) Images showing IL-10 immunohistochemistry in the placenta. *p* < 0.001 (***).

Finally, gene and protein expression of IL-10 in the placenta of women with CVD were analyzed in our study. Our results demonstrate that pregnant women with CVD exhibit decreased gene expression of IL-10 in their placental tissue (Figure 4A; CVD = 16.616 (6.617–23.611), HC = 20.083 (10.611–39.749), *** *p* < 0.0001). Histological examination also suggests that women with CVD had significantly decreased levels of IL-10 protein expression in their placentas (Figure 4B; CVD = 1.000 (0.250–2.000), HC = 1.750 (1.000–3.000), *** *p* < 0.001). According to histopathology pictures, IL-10 protein expression was considerably lower in the placentas of women with CVD, with a punctual expression in the syncytiotrophoblast and inner layers (Figure 4C,D).

## 4. Discussion

The immune system and the inflammatory response play a critical role in normal and pathological pregnancies [24,25,26]. The placenta is a major immunomodulatory organ during pregnancy, determining immune recruitment, education, activation, and function [27]. Physiologically, both the first and the third trimester of pregnancy are associated with a pro-inflammatory status orchestrated by the different maternofetal structures, whereas the second trimester is mostly linked to anti-inflammatory events [28]. However, under obstetric complications like pre-eclampsia, an exacerbated inflammatory response can be observed in both the maternofetal structures and at a systemic level [29]. CVD is associated with mechanical, hormonal, and hemodynamical changes necessary for pregnancy success. Previous works have evidenced that CVD itself is associated with systemic inflammation [1,30]. We previously observed that CVD during pregnancy is also related to inflammatory changes in maternofetal structures [16], as well as to systemic inflammatory changes [15]. In this study, we have reported an increased expression of AIF-1, IL-12A, and IL-18 in the placentas of women with CVD, along with a marked decrease in IL-10. These changes help us to gain further insights into the pathophysiological role of inflammation in the maternofetal structures of women suffering from CVD during pregnancy. 

First, the observed elevation of AIF-1 expression in the placentas of women with chronic venous disease (CVD) suggests a potential link between the inflammatory milieu characteristic of CVD and placental molecular alterations. AIF-1 is a molecule with multiple tissue functions. In more detail, AIF-1 presents both immune and non-immune functions. Some of the immune functions include the regulation of the activation and function of macrophages, T lymphocytes, and dendritic cells, whereas the non-immune activity includes different processes like membrane ruffling, regulation of cell cycle in vascular smooth muscle cells, and endothelial activation [12]. Previous work has demonstrated that AIF-1 is activated in response to diverse inflammatory mediators, including interferon-gamma (IFN-γ), IL-1β, tumor necrosis factor α (TNF-α), β-Estradiol, and IL-2 [13]. Interestingly, altered circulating levels of IFN-γ, IL-1β, TNF-α, and IL-2 have been demonstrated in pregnant women with CVD [15], whereas levels of β-estradiol seem to be associated with increased venous distensibility and CVD development [31], although the levels of this hormone have not been studied in this population yet. Therefore, it is possible that AIF-1 overexpression in the placenta of women with CVD might be a consequence of the systemic inflammatory environment observed in these patients. In turn, AIF-1 is associated with an altered expression in other inflammatory cytokines [11] and also modulates the expression of different chemokines, hence inducing chemotaxis of circulating monocytes [32]. To the best of our knowledge, no studies have evaluated AIF-1 expression either in the placental tissue of healthy or pathological pregnancies. As we show in this study, AIF-1 expression is observed in the placenta of both non-pathological and CVD pregnancies, demonstrating that this molecule can be important in the regulation of physiological pregnancies but also that its dysregulation may contribute to the inflammatory environment associated with CVD. However, further research is warranted to deepen our understanding of the relationship between AIF-1 overexpression and possible adverse pregnancy outcomes in individuals affected by CVD, which will, in turn, allow for evaluation of the possible prognostic value and potential therapeutic implications of AIF-1. 

Additionally, investigating the downstream effects of AIF-1 overexpression, particularly its influence on the expression of inflammatory cytokines and chemokines, might illuminate the pathways linking its dysregulation to the recruitment and activation of immune cells within the placenta. In this sense, our findings revealed a concomitant decrease in the expression of IL-10 together with elevated levels of IL-12A and IL-18. IL-10 is a major anti-inflammatory cytokine involved in the limitation of host immune responses, aiding in the maintenance of tissue homeostasis [33]. Prior work has found that IL-10 tends to be downregulated in the placenta in a gestational age-dependent manner, and that this mechanism is critical for the underlying mechanism of parturition [34]. However, a greater decrease in this cytokine in the placentas of women with distinct placental pathologies, like pre-eclampsia, recurrent spontaneous abortion (RSA), and preterm birth, has also been described [35,36,37,38]. Likewise, we previously reported a marked decrease in gene and protein expression of IL-10 in the umbilical cord of women with CVD [14], supporting a potential relationship between altered patterns of cytokine expression and obstetric complications. In regard to the possible role of IL-10 in the placentas of women with CVD, previous work has related IL-10 downregulation to hypoxic events [39], oxidative stress [40], and changes in matrix metalloproteinase 9 (MMP-9) expression [41]. Importantly, we previously observed the implication of all these mechanisms in the placentas of women with CVD [29]. Collectively, our results suggest a potential association of IL-10 downregulation with the underlying mechanisms driving CVD-related obstetric complications.

Conversely, both IL-12A and IL-18 are two cytokines more involved in pro-inflammatory events. IL-12A (also named p35) is a subunit of the heterodimeric cytokine, together with IL-12B (also designated p40). It is produced by B cells, dendritic cells, and macrophages, and regulates IFN-γ production, T cell differentiation, and function [42]. IL-18 is a proinflammatory cytokine, belonging to the IL-1 family, that is involved in Th1, NK cells, Th2, IL-17-producing γδ T cells, and macrophage activation [43]. Both IL-12 and IL-18 are involved in IFN-γ production by Th1 cells, although IL-12 is also a differentiation factor that induces Th1 polarization, and IL-18 shows a tight relationship with the NLRP3 inflammasome activation [44]. Both IL-12A and IL-18 expression has been demonstrated in the placenta under physiological pregnancies [45,46], whereas, in pathological conditions, a significant increase in IL-18 [47,48] but not in IL-12A [49] expression has been reported in previous works. We previously observed that IL-12A and IL-18 were overexpressed in the umbilical cord of women with CVD [14], and that they were also augmented in the serum of the women affected by this condition, as well as in their newborns [15]. The increased gene and protein expression of both cytokines in the placenta of women with CVD are in line with these results, although our results might be taken cautiously, especially in regard to IL-12A expression. Future works should explore the expression levels of IL-12 in patients with CVD and evaluate the pathophysiological consequences of IL-18 overexpression. In particular, due to the association of this cytokine with the NLRP3 inflammasome—a system commonly increased in pathological pregnancies [50,51]—further efforts might be directed to explore this component in women with CVD. 

## 5. Conclusions

**Figure 5:**
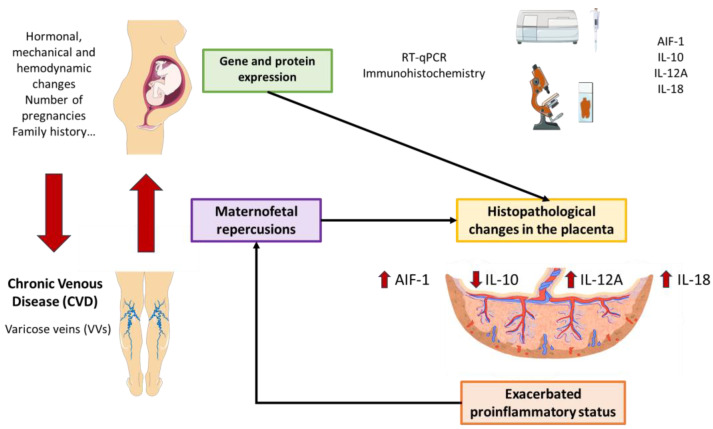
Graphic summary of the results.

In this work, we have observed a notable increase in AIF-1, IL-12, and IL-18 expression in the placentas of women with CVD, together with a marked decrease in the anti-inflammatory cytokine IL-10. As summarized in Figure 5, future works should aim to evaluate the pathological consequences of these changes, thus aiding in understanding the precise relationship between CVD and maternofetal wellbeing.

## Figures and Tables

**Table 1:** Demographic and clinical features. Healthy control, or HC, compared to women diagnosed with chronic venous disease. This table corresponds to the same sample of patients also studied in previous works [9].

	CVD (*n* = 62)	HC (*n* = 52)
Median age (IQR), years	33 (22–40)	34 (27–41)
Median gestational age (IQR), weeks	40.5 (39–41.5)	41 (39–42)
C-section delivery, *n* (%)	12 (19.4)	9 (17.3)
Delivery via vagina, *n* (%)	50 (80.6)	43 (82.7)
CVD (CEAP), *n* (%)		
CEAP 1	37 (59.7)	0 (0)
CEAP 2	21 (33.8)	0 (0)
CEAP 3	4 (6.5)	0 (0)
Prior pregnancies, *n* (%)	33 (53.2)	19 (36.5)
Prior abortions, *n* (%)	14 (22.6)	9 (17.3)
Regular periods of menstruation, *n* (%)	50 (80.6)	42 (80.7)
Profession of inactivity, *n* (%)	41 (66.1)	40 (76.9)

## Data Availability

The data used to support the findings of the present study are available from the corresponding author upon request.

## Supplementary Materials

The following supporting information can be downloaded at https://www.mdpi.com/article/10.3390/jpm14010087/s1. Table S1: Primers used for RT-qPCR: sequences and binding temperatures (Temp); Table S2: Primary and secondary antibodies used and their dilutions.
