# Supplementary material for: Histopathological Clues of Enhanced Inflammation in the Placental Tissue of Women with Chronic Venous Disease in Lower Limbs during Pregnancy
Source: J Pers Med. 2024 Jan 12;14(1):87. doi: 10.3390/jpm14010087 (PMC10821220; doi:10.3390/jpm14010087)
Supplement: Supplementary file 1 [file jpm-14-00087-s001.zip › jpm-2783623-supplementary.pdf]

Table S1. Primers used for RT-qPCR: sequences and binding temperatures (Temp).

GENE	SEQUENCE Fwd (5´→3´)	SEQUENCE Rev (5´→3´)	Temp
TBP	TGCACAGGAGCCAAGAGTGAA	CACATCACAGCTCCCCACCA	60ºC
AIF-1	TGAAAACCCTCCAGTCAGCG	GTCAGGGTAGCTGAACGTCT	60 °C
IL-12A	GCACAGTGGAGGCCTGTTTA	GCCAGGCAACTCCCATTAGT	60.2 °C
IL-18	GCTGAAGATGATGAAAACCTGGA	GAGGCCGATTTCCTTGGTCA	59.5 °C
IL-10	TGCTCTTGCAAAACCAAACCA	GGGAGGTCAGGGAAAACAGC	60 °C

Table S2. Primary and secondary antibodies used and their dilutions.

Antigen	Species	Dilution	Provider	Protocol Specifications
AIF-1	Goat polyclonal	1: 50	Abcam (ab5076)	EDTA pH = 9 before incubation with blocking solution
IL-12A	Rabbit monoclonal	1:100	Abcam (ab131039)	EDTA pH = 9 before incubation with blocking solution
IL-18	Rabbit monoclonal	1:200	Abcam (ab243091)	10 mM Sodium citrate pH = 6 before incubation with blocking solution
IL-10	Rabbit Polyclonal	1:250	Abcam (ab217941)	100% Triton 0.1% in PBS, 10 min, before incubation with blocking solution
IgG (Rabbit)	Mouse	1:1000	Sigma-Aldrich (RG-96/B5283)	------
IgG (Goat)	Mouse	1:100	Sigma-Aldrich [GT- 4/B3148]	------
